# Transcriptome analysis of yellow passion fruit in response to cucumber mosaic virus infection

**DOI:** 10.1371/journal.pone.0247127

**Published:** 2021-02-24

**Authors:** Lijuan Chen, Donglei Sun, Xingxing Zhang, Danqing Shao, Yinglin Lu, Yuxing An

**Affiliations:** 1 Institute of Bioengineering, Guangdong Academy of Sciences, Guangzhou, Guangdong, China; 2 Key Laboratory of Natural Pesticides and Chemical Biology, Ministry of Education, South China Agricultural University, Guangzhou, Guangdong, China; CSIR- Institute of Himalayan Bioresource Technology, INDIA

## Abstract

The cultivation and production of passion fruit (*Passiflora edulis*) are severely affected by viral disease. Yet there have been few studies of the molecular response of passion fruit to virus attack. In the present study, RNA-based transcriptional profiling (RNA-seq) was used to identify the gene expression profiles in yellow passion fruit (*Passiflora edulis* f. *flavicarpa*) leaves following inoculation with cucumber mosaic virus (CMV). Six RNA-seq libraries were constructed comprising a total of 42.23 Gb clean data. 1,545 differentially expressed genes (DEGs) were obtained (701 upregulated and 884 downregulated). Gene annotation analyses revealed that genes associated with plant hormone signal transduction, transcription factors, protein ubiquitination, detoxification, phenylpropanoid biosynthesis, photosynthesis and chlorophyll metabolism were significantly affected by CMV infection. The represented genes activated by CMV infection corresponded to transcription factors WRKY family, NAC family, protein ubiquitination and peroxidase. Several DEGs encoding protein TIFY, pathogenesis-related proteins, and RNA-dependent RNA polymerases also were upregualted by CMV infection. Overall, the information obtained in this study enriched the resources available for research into the molecular-genetic mechanisms of the passion fruit/CMV interaction, and might provide a theoretical basis for the prevention and management of passion fruit viral disease in the field.

## Introduction

Passion fruit (*Passiflora edulis*) is widely cultivated throughout tropical and subtropical regions of the world. However, the cultivation and production of passion fruit are severely affected by various pathogens, such as viruses, bacteria and fungi. Among them, viral disease is an extremely serious disease of passion fruit and a yield-limiting factor for the crop. The causal agents of passion fruit viral disease are various, including members of the genus *Potyvirus* [1–5], *Cucumovirus Begomovirus* [6], *Tymovirus* [7], *Cilevirus* [8], *Carlavirus* [9] and *Begomovirus* [10]. Passion fruit woodiness virus (PWV, genus *Potyvirus*) is the most economically important viral disease of passion fruit plants in Brazil, which can significantly affect the content of phenolic compounds in rinds [11]. Cowpea aphid-borne mosaic virus (CABMV, genus *Potyvirus*) is another important viral disease of passion fruit plants, which was reported in all of the major producing states in Brazil [12], and also was identified in Africa (Uganda) [13]. Besides, viral disease of passion fruit plants caused by telosma mosaic virus (TeMV, genus *Potyvirus*) has been reported in Thailand and some areas of China [14–16]. Recently, we found that the presence of the *geminiviruses* ramie mosaic virus (RamMV) in passion fruit in China [17].

As the type member of the *Cucumovirus* genus, cucumber mosaic virus (CMV) has a worldwide distribution and a very wide host range, causing many diseases in plants. The occurrence of CMV infection on yellow passion flower plants was apparently first identified in New South Wales, Australia [18]. Since then, the isolation of CMV from *Passiflora* species was reported in Japan [19], Brazil [20], as well as in Italy [21]. Pares et al. (1985) suggested that a “tip necrosis” disease on Australian passion fruit was associated with dual infection with CMV and PWV which kills the plant but neither of the viruses by alone is so destructive [22]. Besides, we found that CMV, with TeMV and RamMV, existed in passion fruit plants with foliar virus-like symptoms in China [17].

To successfully combat the invasion of a wide range of pathogens, plants have evolved elaborate perception systems and defence strategies. Once pathogens are recognized, plants initiate rapid defence responses and activate numerous signalling pathways, including the generation of reactive oxygen species (ROS); the production of antioxidant enzymes; the expression of pathogenesis-related proteins (PRs); the hypersensitive responses; the establishment of systemic acquired resistance, to against pathogens attack [23–27]. Additionally, plant hormones play pivotal roles in the well-developed defence system of plants with pathogens attack. Salicylic acid (SA), jasmonic acid (JA) and ethylene (ET), are considered to be key players in plant defence system. Besides, many other phytohormones including auxin (AUX), abscisic acid (ABA), cytokinins (CTK), gibberellins (GA), and brassinosteroids (BR), also participate in plant defence against different types of pathogens by crosstalk with the SA-JA-ET cascades [28–31]. Actually, the main defence strategies in plants often vary with differences in the interactions between specific pathogen and their respective hosts. Until now, relatively limited information is available on the defence strategies adopted by passion fruit to protect against pathogen invasion.

The large-scale cultivation of passion fruit has attracted increased attention in recent years. In China, passion fruit has become an important commercial fruit with large-scale cultivation in the southern part of China such as in Guangxi, Guangdong, and Fujian provinces. Besides, compared to purple passion fruit, yellow passion fruit is becoming more and more popular with consumers. Unfortunately, viral disease also has become one of the most severe constraint for the cultivation and production of yellow passion fruit in China. Thus, it is a pressing task to improve yield and quality of fruits from the virus infected orchards. In the present study, transcriptome analysis was used to identify the gene expression profiles in yellow passion fruit plants after CMV inoculation. Our results enriched the resources available for the responses of passion fruit to CMV infection. These evidences also provided a theoretical foundation for further research on the molecular mechanisms of the interaction between passion fruit and viral pathogens.

## Materials and methods

### Plant cultivation and virus inoculation

Yellow passion fruit seeds were cultivated in a temperature-controlled growth chamber at an irradiation dose of 100 μM m^-2^ s^-1^ on a 16-h light/8-h dark cycle with an average temperature of 27°C. After approximately 5 weeks of growth, two leaves from the bottom insertions were mechanically inoculated with CMV isolate AH (CMV-AH), which were acquired from the Ministry of Agriculture Plant Quarantine Institute, China [32]. The virus isolates of CMV-AH were maintained in an aqueous suspension of 0.02 M sodium phosphate buffer (PBS) at 4°C. Corresponding leaves from the control plants were mock-inoculated with PBS. Sample leaves were collected at ten days post inoculation (dpi). Leaf samples were frozen immediately in liquid nitrogen and stored at -80°C until RNA extraction for transcriptome sequencing. At least six parallel leaves from three plants were mixed as one simple. Each sample has three biological replicates.

### Library preparation and transcriptome sequencing

Illumina sequencing was carried out at Biomarker Technologies Co., LTD., in Beijing, China. Equal quantities of RNA from the six samples (control plants, CK1-3; CMV-inoculated, CMV1-3) were prepared for six transcriptome libraries construction. The total RNA was extracted using the Quick RNA isolation kit (Bioteke Corporation, Beijing, China) according to the manufacturer’s recommendations. Then, the degradation and contamination of RNA were monitored on 1% agarose gels. We evaluated the RNA purity using the NanoPhotometer® spectrophotometer (IMPLEN, CA, USA) and the RNA concentration using the Qubit® RNA Assay Kit in a Qubit® 2.0 Fluorometer (Life Technologies, CA, USA), respectively. Moreover, the RNA Nano 6000 Assay Kit of the Agilent Bioanalyzer 2100 system (Agilent Technologies, CA, USA) was adopted to assess the RNA integrity.

Sequencing libraries were generated using the NEBNext® Ultra™ RNA Library Prep Kit for Illumina® (NEB, USA), following the manufacturer’s protocol. Index codes were added to attribute sequences to each sample. First, mRNA was purified from the total RNA using poly-T oligo-attached magnetic beads. Fragmentation was carried out using divalent cations under an elevated temperature in NEBNext First Strand Synthesis Reaction Buffer (5X). Next, the first strand cDNA was synthesized using a random hexamer primer and M-MuLV Reverse Transcriptase (RNase H-). Then, second strand cDNA synthesis was performed using DNA Polymerase I and RNase H. The remaining overhangs were converted into blunt ends via exonuclease/polymerase activities. After adenylation of the 3’ ends of the DNA fragments, the NEBNext Adaptors with hairpin loop structures were ligated to prepare for hybridization. In order to select cDNA fragments of preferentially 150~200 bp in length, the AMPure XP system (Beckman Coulter, Beverly, Brea, FA, USA) was adopted to purify the fragments. Then, 3 μl of the USER Enzyme (NEB, USA) was used with size-selected, adaptor-ligated cDNA at 37°C for 15 min, followed by a 5 min incubation at 95°C before PCR. Next, PCR was performed with the Phusion High-Fidelity DNA polymerase, Universal PCR primers and the Index (X) Primer. Finally, the PCR products were purified (AMPure XP system), and the library quality was assessed using the Agilent Bioanalyzer 2100 system (Agilent Technologies, Palo Alto, CA, USA).

The clustering of the index-coded samples was performed using the cBot Cluster Generation System and the TruSeq PE Cluster Kit v3-cBot-HS (Illumina, USA) according to the manufacturer’s instructions. After cluster generation, the library preparations were sequenced on an Illumina NovaSeq 6000 platform, and 150 bp paired-end reads were generated.

### RNA-seq data analysis

Raw data (raw reads) in fastq format were firstly processed through in-house Perl scripts. Clean data (clean reads) were obtained by removing reads containing adapter sequences, reads containing poly-N, and low-quality reads from raw data. At the same time, the Q30 values, GC-content, and the sequence duplication level of the clean data were calculated. The Phred cutoff of Q30 is 85%. All the downstream analyses were based on high-quality clean data.

The left files (read1 files) from all libraries/samples were pooled into one large left.fq file. The right files (read2 files) were pooled into one large right.fq file. Transcriptome assembly was accomplished based on the left.fq and right.fq using program Trinity (v2.5.1) with min_kmer_cov set to 2 by default and all other parameters set to the default values [33]. Gene functions were annotated based on the following databases: NR (NCBI non-redundant protein sequences); Pfam (Protein family); KOG/COG (Clusters of Orthologous Groups of proteins); Swiss-Prot (A manually annotated and reviewed protein sequence database); KEGG (The Kyoto Encyclopedia of Genes and Genomes); GO (Gene Ontology); eggNOG (evolutionary genealogy of genes: Non-supervised Orthologous Groups).

### Differential expression analysis

Gene expression levels were estimated by RSEM software for each sample [34]. Clean data were mapped back onto the assembled transcriptome using RSEM software, and a read count for each gene was obtained from the mapping results.

For the samples with biological replicates, the differential expression analysis of two groups was performed using the DESeq R package (1.10.1) [35]. DESeq provided statistical routines for determining differential expression in digital gene expression data using a model based on the negative binomial distribution. The resulting *P* values were adjusted using the Benjamini and Hochberg’s approach for controlling the false discovery rate (FDR) [36]. A FDR < 0.01 and fold_change ≥ 2 were set as the thresholds for significant differential expression.

### DEGs functional annotation

GO enrichment analysis of the differentially expressed genes (DEGs) was implemented using the GOseq R packages based the Wallenius non-central hyper-geometric distribution [37], which can adjust for gene length bias in the DEGs. KEGG was used to map sequences to pathways (http://www.genome.jp/kegg/). The KOBAS software was used to test the statistical enrichment of differential expression genes in the KEGG pathways [38].

### Quantitative real-time polymerase chain reaction validation

Validation of the RNA-seq data for nine different genes was performed using quantitative real-time polymerase chain reaction (qRT-PCR) analysis (Bio-Rad Laboratories, Hercules, CA, USA). The cDNA was amplified using SYBR Premix Ex Taq (TaKaRa Bio, Inc., Dalian, China). The amplification of the target genes was monitored every cycle based on SYBR green I fluorescence. Each reaction was performed in triplicate for each of the three biological replicates. The amplification of the ribosomal RNA small subunit methyltransferase (c32915.graph_c0) was used as an internal control. The relative expression levels of the selected genes were determined using the comparative *C*_*T*_ method [39]. The primer sequences used in the study are shown in S1 Table.

### Electron microscopy and paraffin sections

The fresh samples were prefixed in a mixed solution of 3% glutaraldehyde, and the electron microscopy was performed as previously described [32]. The sections were examined with a transmission electron microscope (TEM; HITACHI, H-600IV, Japan). Paraffin sections were collected as previously described with several modifications [40].

### Measurement of chlorophyll contents and photosynthesis

The contents of chlorophylls (Chl) a and b were determined according to the methods of Kichtenthaler and Wellburn (1983) [41]. The stomatal conductance, transpiration rate and net CO_2_ assimilation were measured by portable photosynthesis system TPS-2 (PP Systems Company, England), according to the manufacturer’s protocol.

### Oxidative damage estimation

Electrolyte leakage was assayed as previously described [42]. First, approximately 0.2 g of fresh leaves was cut into about 1 cm segments and placed in 5 mL of deionized water at room temperature. Then, the conductivity (*C*_*1*_) of the fresh leaves was measured after 45 min. Next, the samples were boiled at 100°C for 15 min to achieve 100% electrolyte leakage (*C*_*2*_). The relative conductivity of the plasma membranes was measured as (electrical conductivity *C*_*1*_/total electrical conductivity *C*_*2*_) × 100%.

Lipid peroxidation was estimated by measuring the thiobarbituric acid-reactive substance (TBARS). Approximately 0.5 g of fresh leaves was homogenized in 5 mL of 5% (w/v) trichloroacetic acid in an ice bath. Then, the homogenates were transferred into a tube and centrifuged at 1,000×*g* for 10 min at 4°C. The resulting supernatants were used to determine the TBARS content as previously described [43].

### Determination of antioxidant enzymes and phenylalanine ammonia-lyase activities

For the enzyme assays, 0.3 g fresh leaf tissue was ground with 3 mL ice-cold 25 mM HEPES buffer (pH 7.8) containing 0.2 mM EDTA, 2 mM ascorbic acid and 2% (w/v) polyvinylpyrrolidone. Then, the homogenates were centrifuged at 4°C for 20 min at 12,000×*g*, and the resulting supernatants were used to determine the enzymatic activity. The ascorbate peroxidase (APX), catalase (CAT), peroxidase (POD) and superoxide dismutase (SOD) activities were assayed as previously described [42]. Phenylalanine ammonia-lyase (PAL) activity was determined by spectrophotometric measurement of the conversion of L-phenylalanine to cinnamic acid at 268 nm via the modified method of Shirasawa et al. (2012) [44].

### Statistical analysis

The results are expressed as the means of at least three independent measurements. The data were statistically evaluated using standard deviations and one-way analysis of variance (ANOVA). Differences were considered to be statistically significant when *P* < 0.05.

## Results

### Annotation of unigenes in passion fruit

**Fig 1:**
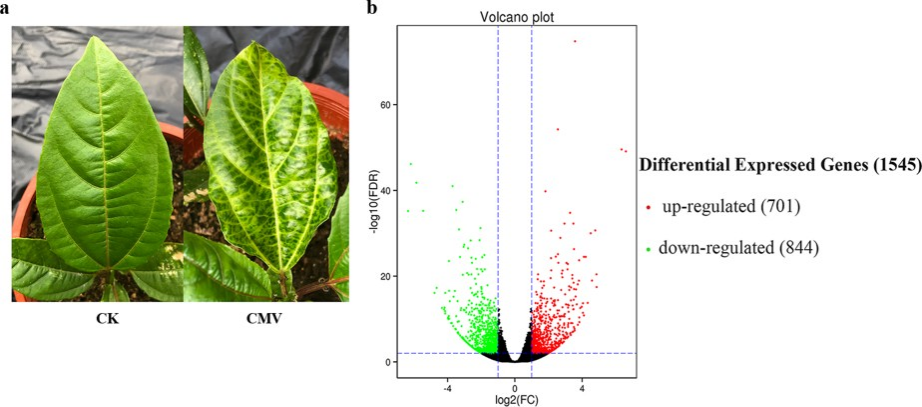
(a) Symptoms of yellow passion fruit leaves systemically infected with cucumber mosaic virus (CMV). (b) Volcano plot of significant differences in gene expression between control (CK) and CMV-inoculated groups (CK1_CK2_CK3_vs_CMV1_CMV2_CMV3), red for upregulated, green for downregulated.

CMV can infect yellow passion fruit plants and give rise to symptoms that included mosaic and yellow spots on systematic leaves (Fig 1A). However, yet there have been few previous studies of the transcriptional responses of the passion fruit host to the viral pathogen. In this study, RNA-Seq was used to profile transcriptional changes in yellow passion fruit leaves after CMV inoculation. Since the genome sequence of passion fruit is not available, we first defined the transcriptome of passion fruit using RNA-Seq. Equal quantities of total RNA from the six samples were separately used for library construction. The libraries were sequenced on an Illumina NovaSeq 6000 platform. A total of 20,492,822–29,184,031 clean reads were obtained using Illumina paired-end sequencing (S2 Table). The cleaned reads were used to assemble the transcripts. A total of 324,518 transcripts were assembled according to average and N50 lengths of 2,689 and 3,583 bp, respectively (S3 Table). The length distribution of transcripts is displayed in S1 Fig. Among transcripts representing the same gene, the longest one was considered as unigene. After gap filling with paired-end reads, the transcripts were assembled into 50,139 unigenes. The mean length of unigenes was 1,006 bp, and the N50 length of unigens was 2,202 bp (S4 Table). The length distribution of unigenes is displayed in S2 Fig. The BLAST results for the unigenes were compared against eight databases, including the COG, GO, KEGG, KOG, Pfam, Swissprot, eggNOG and NR databases. Results indicated that a total of 27,203 unigenes have BLAST results, which are shown in S5 Table.

The unigenes were all subjected to search against the COG database for functional prediction and classification. COG analysis indicated that 8,389 unigenes had COG annotations and were distributed among 26 functional classes (S3 Fig). The largest group was ‘translation, ribosomal structure and biogenesis’ (1032), followed by ‘general function prediction only’ (948), ‘carbohydrate transport and metabolism’ (862), and ‘posttranslational modification, protein turnover, chaperones’ (791).

A total of 18,263 unigenes had GO annotations and were distributed among 50 functional classes (S4 Fig). In the cellular component (CC) category, genes involved in ‘cell’ (8834), ‘cell part’ (8807), ‘membrane’ (6786), ‘organelle’ (6113), and ‘membrane part’ (5292), were highly represented. The molecular function category (MF) mainly included genes involved in ‘catalytic activity’ (9427) and ‘binding’ (8889). The ‘metabolic process’ (9499), ‘cellular process’ (8900), and ‘single-organism process’ (6210) were the dominant biological process (BP) categories.

Unigene metabolic pathway analysis was also conducted using KEGG. 10,229 unigenes with KEGG annotations were distributed in 127 pathways (S5 Fig). The ‘ribosome’ (707), ‘carbon metabolism’ (463), ‘biosynthesis of amino acids’ (381), ‘protein processing in endoplasmic reticulum’ (280), and ‘plant hormone signal transduction’ (264), were the dominant KEGG pathways.

### Identification of DEGs in passion fruit with CMV infection

The transcriptome sequences assembled by Trinity were used as reference sequences [33]. The clean data of each samples were mapped back onto the assembled transcriptome using the RSEM software [34]. The statistics of clean reads mapped to the reference sequences are shown in Table 1. Next, we compared pairs of transcriptome profiles of the control and the CMV-infected samples to identify DEGs. A total of 1,545 DEGs were obtained (CK1_CK2_CK3_vs_CMV1_CMV2_CMV3), of which 701 DEGs were upregulated and 844 DEGs were downregulated in the inoculated sample compared to the control samples (Fig 1B). The upregulated DEGs and downregulated DEGs with annotation are displayed in S1 Data and S2 Data, respectively.

**Table 1:** DGE reads mapped to the reference sequences.

Sample name	Clean Reads	Mapped Reads	Mapped Ratio
CK1	29,184,031	25,574,717	87.63%
CK2	26,439,556	23,061,362	87.22%
CK3	21,850,221	19,221,602	87.97%
CMV1	20,492,822	17,987,883	87.78%
CMV2	21,476,562	18,705,203	87.10%
CMV3	22,132,110	19,257,598	87.01%

### Functional annotation of DEGs in passion fruit with CMV infection

**Fig 2:**
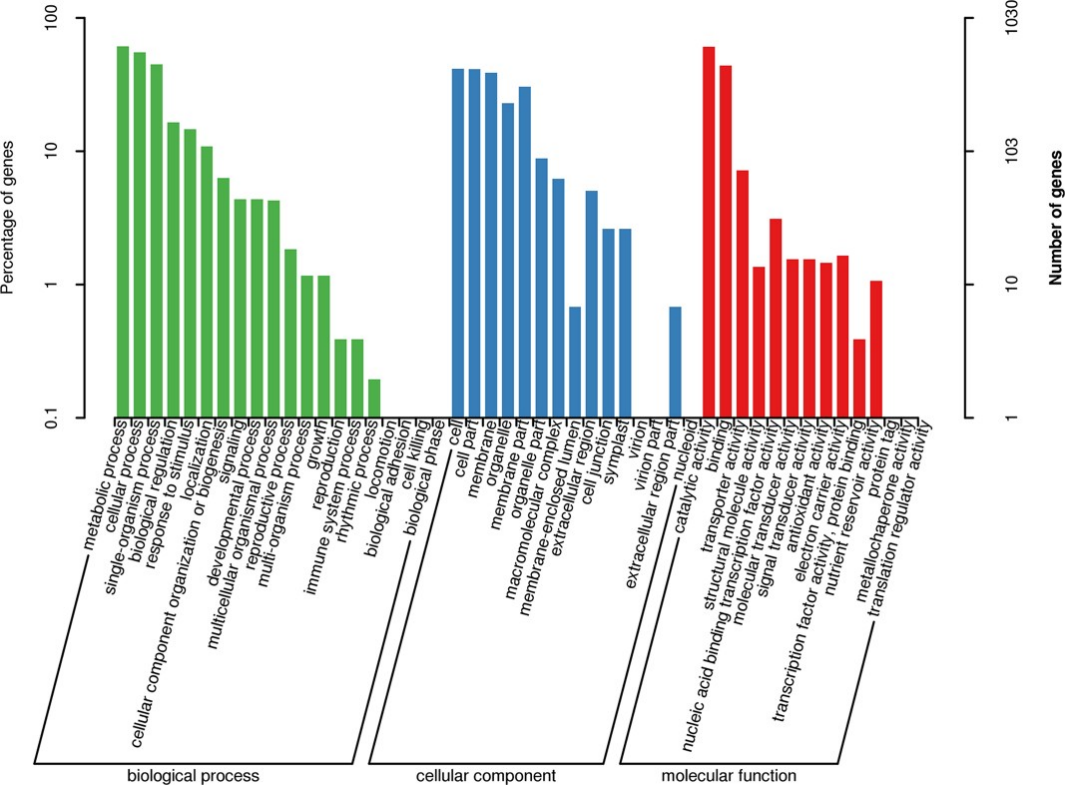
Gene ontology (GO) classification of differentially expressed genes (DEGs). The results are summarized in three main categories: biological process (BP), cellular component (CC), and molecular function (MF).

To obtain a functional categorization of the DEGs, GO analysis was used to classify the DEGs. A total of 1,030 DEGs were categorized into 49 functional groups that were clustered in three main GO classification categories (Fig 2 and S3 Data), with 20 annotations belonging to the BP categories, 15 annotations belonging to the CC categories and 14 annotations belonging to the MF categories. Among 20 different BP categories, metabolic process (630 DEGs), cellular process (568 DEGs), and single-organism process (461 DEGs), were the three most frequently identified terms. Within the CC categories, the major terms were cell (429 DEGs), cell part (424 DEGs) and membrane (400 DEGs). Additionally, catalytic activity, with 625 DEGs, and binding, with 452 DEGs, were dominant in the MF category.

**Fig 3:**
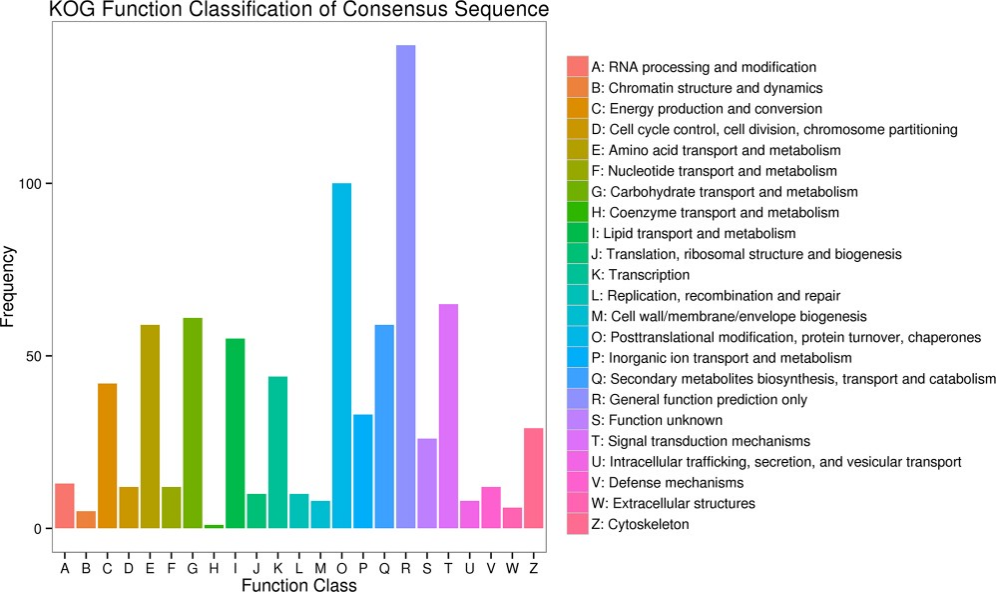
Clusters of eukaryotic orthologous groups (KOG) classification of DEGs.

724 DEGs were assigned to 23 function KOG categories. Among these classifications, the largest group of DEGs was assigned to the general function prediction only, with 140 genes member, followed by posttranslational modification, protein turnover, chaperones (100 DEGs), signal transduction mechanisms (65 DEGs) and carbohydrate transport and metabolism (61 DEGs). Coenzyme transport and metabolism formed the smallest group, with 1 DEGs. The proportions of other classifications ranged from 5 to 59 DEGs (Fig 3 and S4 Data).

**Fig 4:**
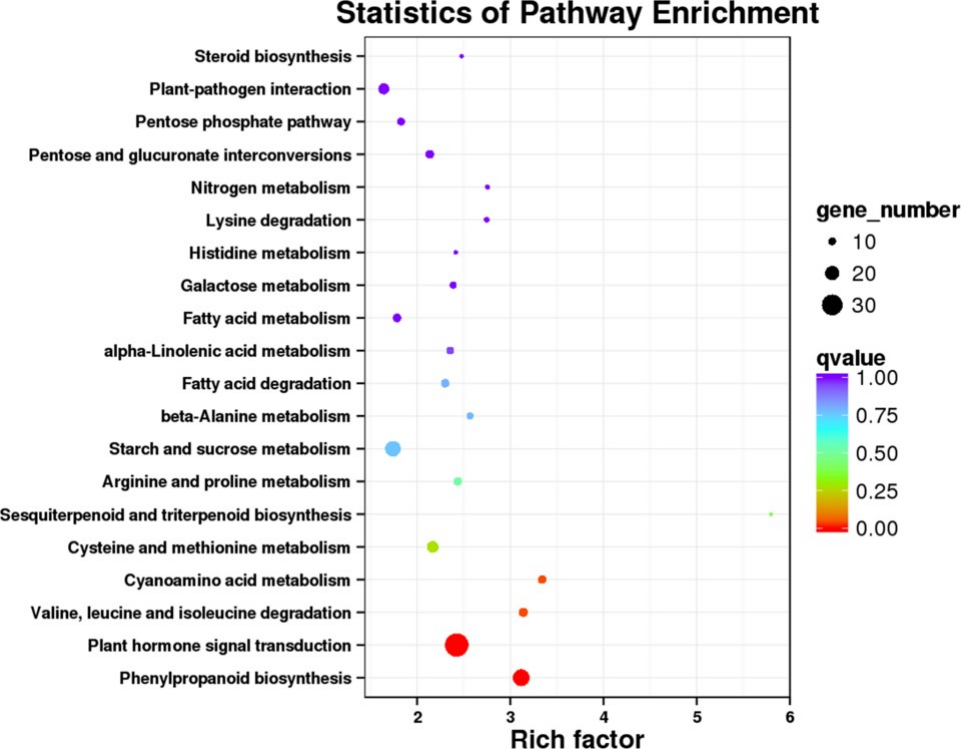
Top twenty Kyoto Encyclopedia of Genes and Genomes (KEGG) pathway enrichment of DEGs.

Additionally, the KEGG database was used to analyze the DEGs. The top 20 enriched KEGG pathways are displayed in Fig 4 and S5 Data. The results indicated that the most frequently represented pathways were ‘phenylpropanoid biosynthesis’ (PATH: ko00940), ‘plant hormone signal transduction’ (PATH: ko04075), ‘valine, leucine and isoleucine degradation’ (PATH: ko00280), and ‘cyanoamino acid metabolism’ (PATH: ko00460), with 24, 34, 12 and 11 gene members, respectively.

### DEGs involved in phytohormone signalling pathways in passion fruit response to CMV infection

**Fig 5:**
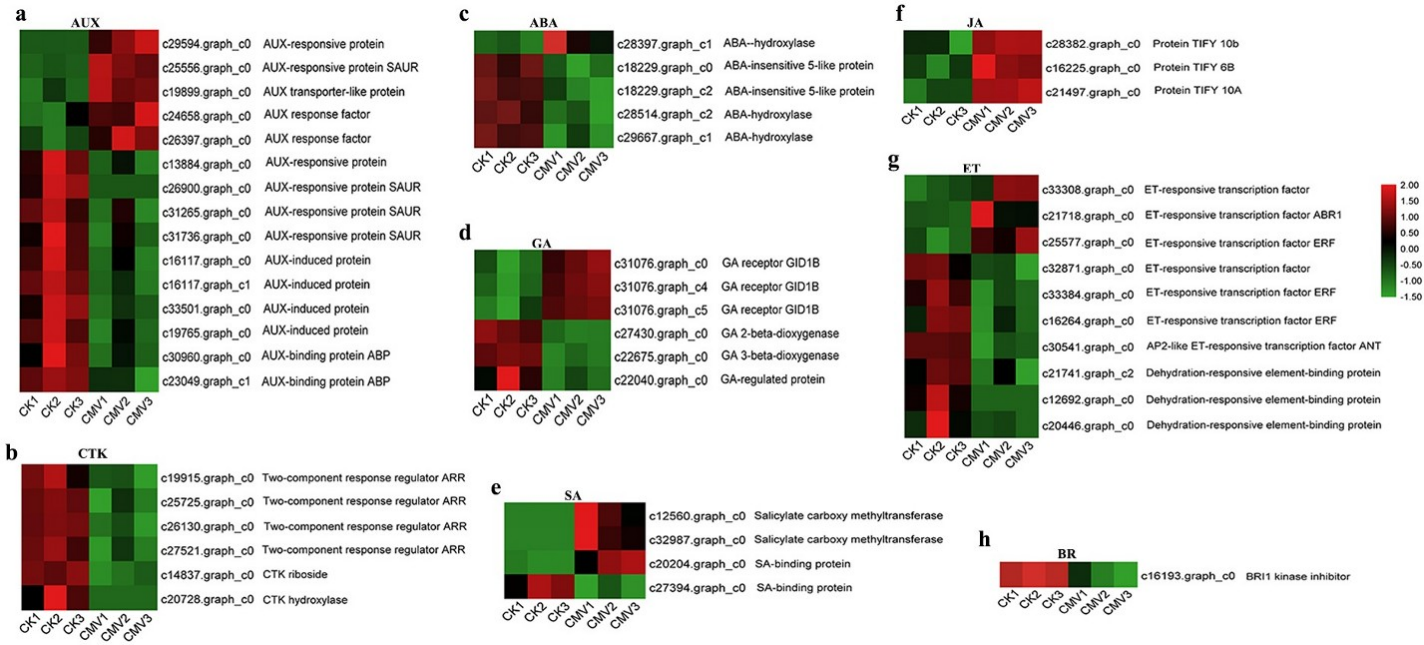
Putative DEGs involved in phytohormone signalling pathways. (a) Auxin (AUX); (b) cytokinins (CTK); (c) abscisic acid (ABA); (d) gibberellins (GA); (e) salicylic acid (SA); (f) jasmonic acid (JA); (g) ethylene (ET); (h) brassionsteroid (BR). Heatmaps were constructed based on the FPKM of DEGs for the six samples. Green to red means the down- to up-regulated level.

Phytohormones are vital regulators of plant-pathogen interactions, and therefore we analyzed the DEGs associated with phytohormones signalling pathways (Fig 5). DEGs involved in AUX signalling including AUX-responsive protein SAUR, AUX-induced protein and AUX-binding protein ABP were differently expressed in CK vs. CMV, and most AUX-related DEGs were notably downregulated after CMV infection (Fig 5A). DEGs involved in CTK signalling, such as two-component response regulator ARR, CTK riboside and CTK hydroxylase were significantly downregulated by CMV infection (Fig 5B). Besides, several ABA- and GA-related genes were differentially expressed in passion fruit response to CMV infection (Fig 5C and 5D). In SA signalling pathway, the genes encoding salicylate carboxymethyl transferase were significantly upregulated by CMV infection (Fig 5E). The genes encoding the jasmonate ZIM domain-containing protein (JA-related genes) were significantly upregulated by virus infection (Fig 5F). Most DEGs in ethylene signalling, such as ethylene-responsive transcription factor ERF, AP2-like ethylene-responsive transcription factor ANT and ethylene-responsive transcription factor TINY-like, were downregulated by CMV (Fig 5G). By contrast, in BR signalling pathway, only one gene encoding the BRI1 kinase inhibitor 1 was found, which was downregulated in passion fruit leaves after virus infection (Fig 5H).

### Transcription factors related to passion fruit response to CMV infection

**Fig 6:**
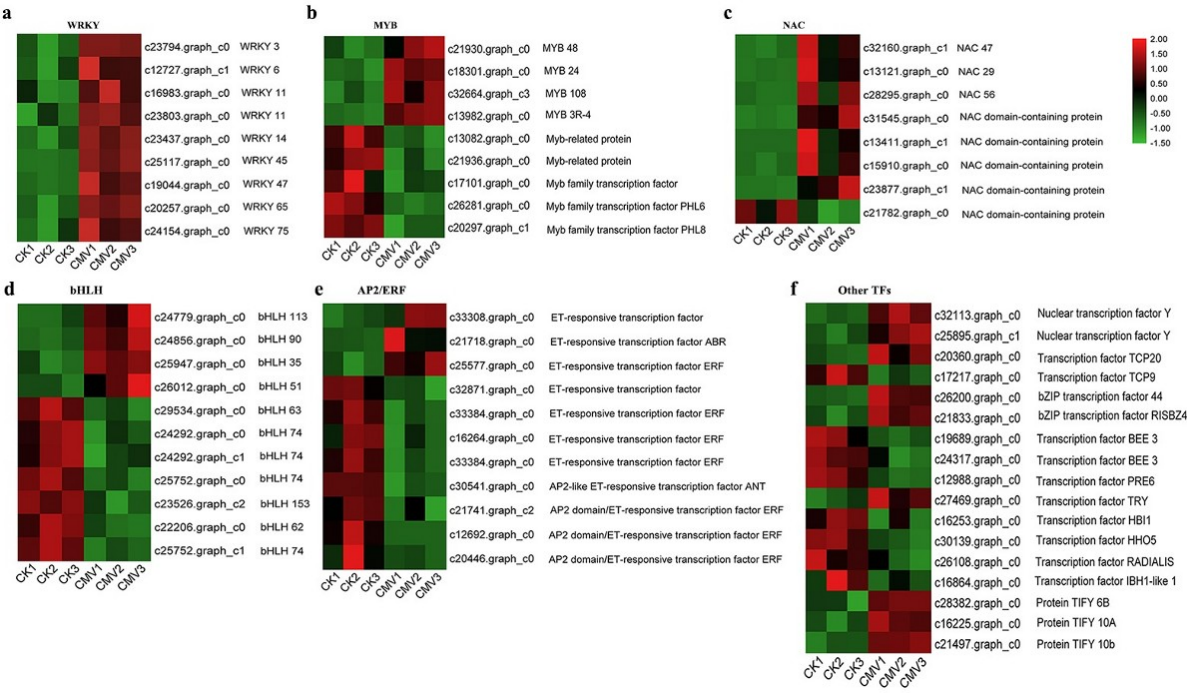
Transcription factors (TFs) in the DEGs sets. Heatmaps were constructed based on the FPKM of DEGs for the six samples. Green to red means the down- to up-regulated level.

Transcription factors (TFs) are concerned as major switches of plant resistance mechanisms under biotic stresses [45]. In the present study, the main TFs families, including WRKY (a conserved N-terminal sequence of WRKYGQK in conjunction with a Zn finger-like motif), MYB (myeloblastosis oncogene), NAC (N-acetyl cysteine), bHLH (basic helix-loop-helix) and AP2/ERF (APETALA2/ET responsive factor) were identified (Fig 6). The most highly represented TFs class was the WRKY family with 9 DEGs, and all members in WRKY family were significantly upregulated by CMV infection (Fig 6A). 7 out of 8 DEGs in NAC family were upregulated by CMV infection (Fig 6C). By contrast, the DEGs belonging to MYB, bHLH and AP2/ERF families displayed different expression patterns (Figs 6C–6E). Besides, other TFs families, such as bZIP (basic region-leucine zipper), nuclear transcription factor Y, and the protein TIFY, were upregulated by virus infection (Fig 6F). These results indicated that the genes from these TFs families may be correlated with the triggering of passion fruit leaves response to CMV attack.

### Ubiquitination related to passion fruit response to CMV infection

**Fig 7:**
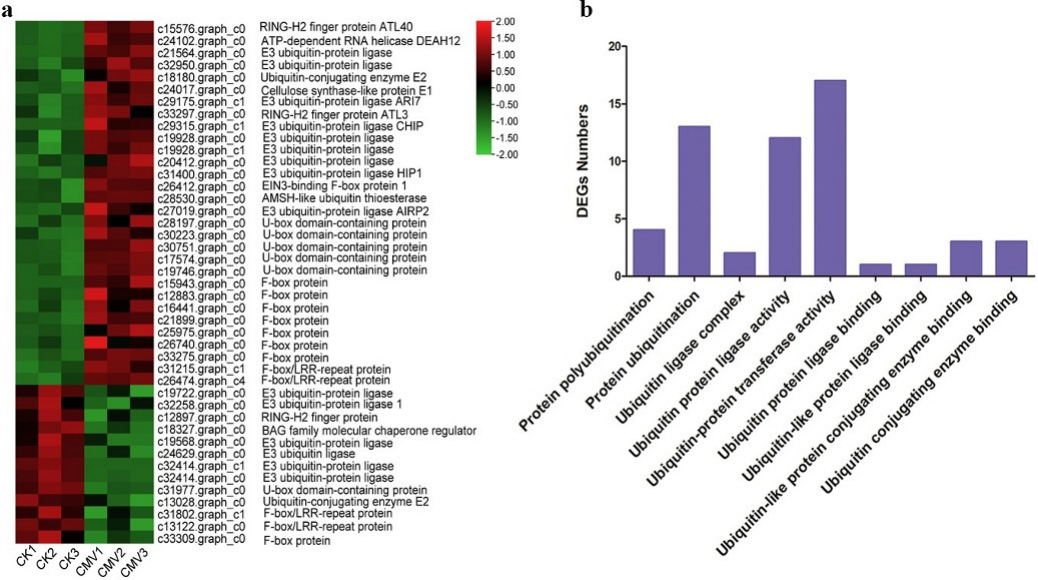
Expression profiling of genes related to protein ubiquitination. (a) Protein ubiquitination in the DEGs sets. Heatmaps were constructed based on the FPKM of DEGs for the six samples. Green to red means the down- to up-regulated level. (b) Numbers of DEGs in different GO terms related protein ubiquitination.

Ubiquitination are also known to play critical roles in regulating plant response to biotic stresses [46]. A total of 43 genes involved in protein ubiquitination were found in the DEGs, including E3 ubiquitin-protein ligase, ubiquitin-conjugating enzyme E2, EIN3-binding F-box protein, and U-box domain-containing protein (Fig 7A). Interestingly, more DEGs were up- than down-regulated in protein ubiquitination pathway of passion fruit. Moreover, the GO analysis indicated that several GO terms related to ubiquitination including protein polyubiquitination (GO:0000209), protein ubiquitination (GO:0016567), ubiquitin ligase complex (GO:0000151), ubiquitin protein ligase activity (GO:0061630), ubiquitin-protein transferase activity (GO:0004842), ubiquitin protein ligase binding (GO:0031625), ubiquitin-like protein ligase binding (GO:0044389), ubiquitin-like protein conjugating enzyme binding (GO:0044390), and ubiquitin conjugating enzyme binding (GO:0031624) were enriched in CK vs. CMV (Fig 7B). Among them, ubiquitin-protein transferase activity (GO:0004842) with 17 DEGs was dominant in the GO terms related to ubiquitination pathway.

### DEGs related to other important stress responses of passion fruit to CMV infection

Phenylpropanoid compounds are natural products derived from cinnamic acid, which is formed from phenylalanine via deamination by the action of phenylalanine ammonia-lyase (PAL), function in plant defence against predators and pathogens [47]. In the present study, KEGG pathways analysis of DEGs showed that the most significant enrichment occurred in “phenylpropanoid biosynthesis” (ko00940), with 24 DEGs (S6 Fig). We measured the activity of PAL, and the results suggested that the activity of PAL significantly increased in CMV-infected plants compared with the CK (S7A Fig). Besides, the functional annotation of DEGs indicated that 19 DEGs were assigned to the “detoxification” GO term, including genes encoding protein detoxification, peroxidase, ascorbate peroxidase, annexin, catalase and glutathione reductase, and most of them were upregulated by CMV infection (S7B Fig). In addition, DEGs encoding wound-induced protein in passion fruit were induced by CMV (S7C Fig). Furthermore, three DEGs encoding PRs were upregulated by CMV (S7D Fig). RNA-dependent RNA polymerases (RDRs) play important role in plant antiviral defence, and the expression of RDR1 and RDR5 were also significantly upregulated by virus infection (S7E Fig). These results implied that these important stress responses may also participate in passion fruit response to CMV infection.

### Changes in physiological characteristics in passion fruit leaves caused by CMV infection

**Fig 8:**
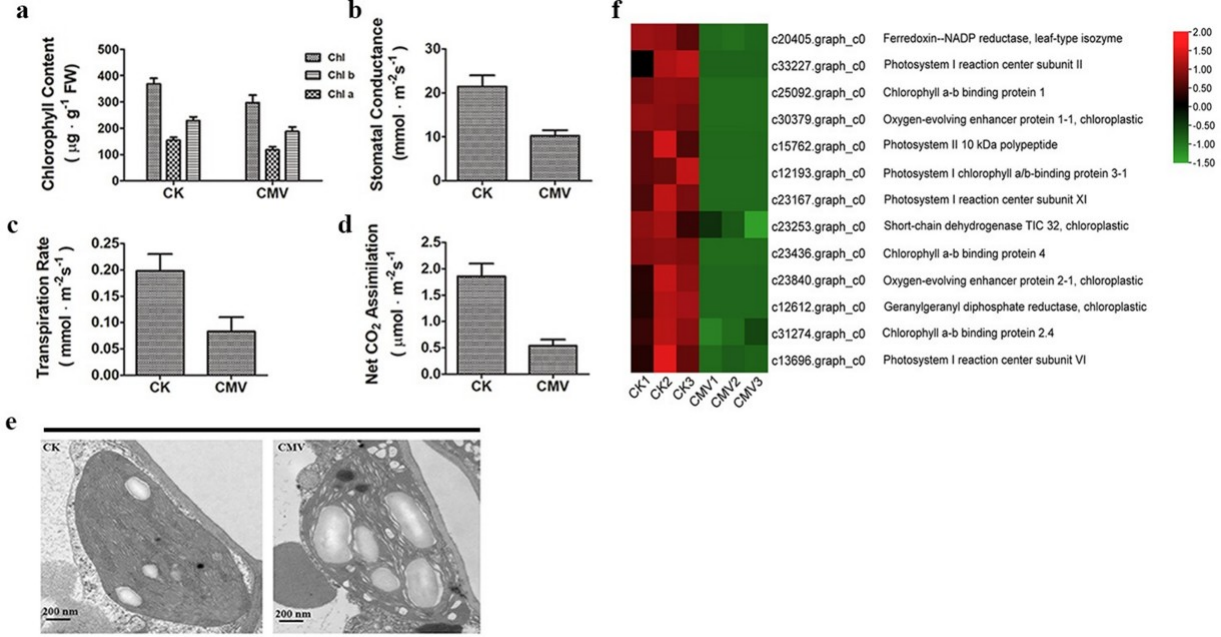
The effects caused by CMV inoculation on photosynthetic systems of passion fruit. (a) Putative DEGs involved in photosynthesis and photosystem. Heatmaps were constructed based on the FPKM of DEGs for the six samples. Green to red means the down- to up-regulated level. (b) Chlorophyll contents; (c) Stomatal conductance; (d) Transpiration rate; (e) Net CO_2_ assimilation; (f) The morphologies of chloroplastids. The scale bars indicate 200 nm.

Physiological characteristics of plants, including chlorophyll contents, photosynthesis and cell damage as parameters to evaluate plants response to pathogens attack. Our results indicated that the level of chlorophyll in passion fruit leaves were significantly decreased by CMV infection (Fig 8A). Additionally, the stomatal conductance, transpiration rate and net CO_2_ assimilation were also depressed by CMV infection (Fig 8B–8D). As the basis of photosynthesis, chloroplasts in passion fruit leaves with CMV infection exhibited incompact granas, and some morphological abnormalities compared to the CK under electron microscopes (Fig 8E). RNA-seq analysis indicated that a substantial number of genes involved in photosynthesis-related processes and chloroplast components were downregulated by CMV infection (Fig 8F). In addition, the cell damage suggested by the relative electrolyte leakage and the TBARS contents, which significantly increased in CMV-infected leaves (S8A and S8B Fig). Moreover, paraffin sectioning showed that the histology within CMV-infected leaves was distinct compared to the CK (S8C Fig). For instance, compared with CK, CMV-infected leaves had relatively few cell layers. Besides, the spongy mesophyll cells in CMV-infected leaves were irregular and replaced by large swollen vacuoles. These results indicated that CMV-infection caused serious damage on the physiological characteristics and structures of passion fruit leaves.

Under biotic stress, plant disease resistance is largely dependent on the initiation of the ROS and ROS scavenging enzymes [48]. Therefore, we measured the activities of APX, CAT, POD and SOD in different groups. The results indicated that the activities of APX, CAT, POD and SOD were obviously induced by CMV infection (S9A–S9D Fig). RNA-seq analysis indicated that a total of 15 DEGs involved in encoding ROS scavenging enzymes in CK vs. CMV, and most of ROS scavenging enzymes-related genes were upregulated by CMV infection (S9E Fig). These results suggested that CMV infection seriously affected the antioxidant systems of passion fruit.

### Validation of RNA-seq data by qRT-PCR

Validation of the RNA-seq data for nine DEGs with annotations was performed via qRT-PCR: probable WRKY transcription factor 47 (WRKY47, c19044.graph_c0), NAC domain-containing protein 100 (NAC100, c28295.graph_c0, c23877.graph_c1), wound-induced protein WIN1 (WIN1, c28696.graph_c1), hypothetical protein POPTR (POPTR, c16955.graph_c0), auxin-responsive protein SAUR20 (SAUR20, c26900.graph_c0), photosystem I reaction center subunit II (PsaD, c33227.graph_c0), elongation factor 1-alpha (ELF1α, c22823.graph_c0), and gibberellin 2-beta-dioxygenase 2 (GA20X2, c27430.graph_c0). Both the qRT-PCR and RNA-seq analysis generally showed similar expression patterns of up- and down-regulation of these DEGs (S10 Fig), which indicated that the changes in expression detected by RNA-seq were valid.

## Discussion

Viral disease is one of the most serious disease of passion fruit, yet there have been few previous studies of the molecular response of the passion fruit to the viral pathogens that might provide information about the molecular-genetic mechanisms of the passion fruit/virus interaction. RNA-seq has been widely applied in research into the interaction between some plants and viral pathogens, but none applied in passion fruit response to virus infection. In the present study, the gene expression profiles between yellow passion fruit plants subjected to CMV infection and the growth without virus were compared, a total of 1,545 DEGs were identified. The identification of gene responses of passion fruit to the CMV infection is an important first step for insights on the molecular mechanism for the interaction between passion fruit and CMV.

There are few resources about how passion fruit response to virus attack and what kinds of effects will caused by these challenges. The relative electrolyte leakage and TBARS contents representing the cell damage degree of plants under stresses were significantly increased by CMV infection (S8 Fig), indicated that CMV infection caused serious cell damages on passion fruit plants. The histology analysis also suggested that CMV infection might have unfortunate effects on the development and growth of passion fruit plants (S8C Fig). As the key components of plant photosynthesis, chloroplasts were seriously damaged by virus infection (Fig 8). A substantial number of genes involved in the photosynthesis-related processes and chloroplast components were downregulated in passion fruit by CMV infection (Fig 8), which is somewhat consistent with the previous suggestion that virus infection can cause the damage on photosynthesis and chloroplasts in plants [32].

Many studies have confirmed that antioxidative metabolisms are implicated in the defence response of plants. For instance, an enhanced CAT activity, with increased contents of total polyphenol and proline, were observed in flax resistant to powdery mildew [49]. POD was involved in pepper yellow mosaic virus resistance in *Capsicum baccatum var*. *pendulum* [25]. Zehra et al (2017) reported that the synergistic effects of SA, MeJA and *Trichoderma harzianum* on enhanced induction of antioxidant defence system like the activities of CAT, APX and ascorbic acid in tomato against Fusarium wilt disease [50]. Our results indicated that CMV infection significantly increased the activities of ROS scavenging enzymes (eg. APX, CAT, POD and SOD) in passion fruit plants (S9 Fig). Several genes encoding ROS scavenging enzymes including POD, CAT, glutathione S-transferase were also upregulated by CMV infection. Especially, 6 out of 8 DEGs encoding POD were upregulated (S9 Fig). These results suggested that antioxidative metabolisms be involved in passion fruit response to CMV, and high levels of antioxidant enzymes activities and related-genes expression are important for passion fruit plants response to CMV.

Phenylpropanoids belong to the largest group of secondary metabolites produced by plants under biotic or abiotic stresses, which is formed from phenylalanine via deamination by the action of PAL. It is thought that the molecular basis for the protective action of phenylpropanoids in plants is their antioxidant and free radical scavenging properties [47]. Some studies demonstrated that PAL genes were essential for plants defence response to pathogens by regulating endogenous SA signalling [51, 52]. In the present study, KEGG pathways enrichment analysis of DEGs showed that the most significant enrichment occurred in “phenylpropanoid biosynthesis” (ko00940) with 24 DEGs (Fig 4 and S5 Data). KEGG pathways related to phenylpropanoid were also identified, including phenylalanine metabolism (ko00360), phenylalanine, tyrosine and tryptophan biosynthesis (ko00400), and flavonoid biosynthesis (ko00941). Additionally, the activity of PAL was increased by virus infection (S7A Fig). These results suggested that phenylpropanoids might be involved in passion fruit response to CMV attack, providing some possible directions for our further research.

As essential components for plants balance stresses and fitness, plant hormones influence different growth and developmental process, as well as defence strategies in plants. Usually, SA, JA and ET are primarily involved in plant defence mechanisms. By contrast, AUX, CTK, GA, ABA and BR also contribute to plant defence responses but key roles in plant development and physiological processes [29–31]. For instance, CTK play key role in the regulation of the active growth, source-sink relationships, metabolism and development in plants. Base on the KEGG pathway analysis, one notable outcome was that the KEGG pathway “plant hormone signal transduction” (ko04075) was significantly enriched (Fig 4 and S5 Data). The KEGG pathway related to zeatin biosynthesis (ko00908) was identified after CMV infection (S11 Fig), and several genes related to CTK biosynthesis were also significantly downregulated by CMV (Fig 5B). Previous researches have suggested that plant-virus interactions retard plant growth through rapid alterations in phytohormones and their signalling pathways [53]. Therefore, we proposed that the differential expression patterns of genes related to AUX, CTK, GA and ABA signalling pathways may has a close relationship with the depressed growth of passion fruit caused by CMV. Meanwhile, it is also need to consider that these phytohormones participate in plant defence against pathogens by crosstalk with the SA-JA-ET cascades, and how they well orchestrate the growth and defence of passion fruit plants after virus infection are need to be clarified in future study.

Various research has identified that a complex positive role of SA in plants against viral pathogens [53–55]. In the present study, functional annotation of DEGs against databases suggested that several DEGs associated with salicylate carboxymethyl transferase were strongly up-regulated in passion fruit after CMV infection (Fig 5E). Besides, the genes encoding jasmonate ZIM domain-containing protein, which are generally classified as negative regulators of JA signalling, were upregulated by CMV (Fig 5F). Most of genes encoding ET-responsive transcription factor were down-regulated by CMV (Fig 5G). Usually, SA is thought to mediate defence signalling in response to biotrophic and hemibiotrophic pathogens, while JA and ET are always associated with defence responses to necrotrophs [56]. However, in order to proper growth, development and best against enemies, plant hormones do not act in isolation but by synergistic or antagonistic crosstalk, and acclimate to each other biosynthesis or responses, leading to fine-tuning of the complex defence response [28, 30]. Based on the previous studies on plant hormones in other plants, we proposed that the SA-, JA- and ET-related signalling pathways might play important roles in passion fruit response to CMV infection.

Previous studies have been demonstrated that TFs families including WRKY, bZIP, MYB, MYC, NAC, bHLH and AP2/ERF participated plants response to biotic stress by regulating gene expression at the transcriptional level, and controlling the activities of these factors to alter the transcriptome of the plants, leading to metabolic and phenotypic changes in plants response to stress [57]. Usually, TFs encoding genes may be differentially regulated (up or down) by different stresses and substantial overlap occurred in the defence pathways allowing integration of different defence signal to fine tune the plant defence against pathogen attack [58]. Moreover, the roles of TFs in plant defence response based on the type of plant-pathogen interaction systems. In the present plant-pathogen system, CMV infection was associated with the altered regulation of TFs belonging to diverse families, such as WRKY, MYB, NAC, bHLH, AP2/ERF, bZIP and TIFY families (Fig 6). Among them, WRKY, MYB, NAC, bHLH, and AP2/ERF families are the main TFs families affected by CMV infection. Additionally, considering the genes, proteins or other compounds in plants induced by pathogens are not only bring a series of effects on the host, but also could affect the pathogen. For instance, Chung et al (2014) reported that the transcription factor AtTIFY4B can interact with *begomovirus* AL2 protein to impact pathogenicity of virus on *Arabidopsis* [59]. Therefore, the exact roles of these regulatory factors in passion fruit/CMV interaction could be interesting studies, and are need to be further investigated.

Increasing evidence supports that ubiquitin and ubiquitination play critical role in plant defence response to various pathogens. For instance, cryptochrome 2 and phototropin 2 regulated resistance protein-mediated viral defence by negatively regulating an E3 ubiquitin ligase in *Arabidopsis* [60]. Lee et al (2011) found that the pepper E3 ubiquitin ligase RING1 gene, *CaRING1*, was required for cell death and the SA-dependent defence response in pepper, and overexpression of *CaRING1* in Arabidopsis confers enhanced resistance to hemibiotrophic *Pseudomonas syringae* pv *tomato* and biotrophic *Hyaloperonospora arabidopsidis* infections [61]. Recently, Han et al (2019) found that the apple U-box E3 ubiquitin ligase *MdPUB29* contributed to activate plant immune response to the fungal pathogen *Botryosphaeria dothidea* possibly by regulating the SA pathway [62]. Here, 43 genes involved in protein ubiquitination were identified exhibiting differential expression. GO analysis identified several GO terms related to ubiquitin and ubiquitination (Fig 7). We proposed that the altered expression levels of ubiquitination-related pathway by CMV infection might is responsible for passion fruit plants with the regulatory potential to fine-tune their defense reactions.

Except for the above mentioned genes and signal pathways in passion fruit plant affected by CMV infection. Some other important defence-related genes also be found in this pathosystem. For instance, PRs are considered as major weapons in plants defence tactics against pathogens [63], and several DEGs encoding PRs were upregulated by CMV infection (S7D Fig). Moreover, previous studies indicated that RDRs play important roles in plant antiviral defence [64, 65], and several DEGs encoding RDRs were significantly upregulated by CMV infection (S7E Fig). These results suggested that the PRs and RDRs may participate in passion fruit antiviral response.

Overall, this study is the first transcriptome study to analyze how passion fruit response to CMV infection. The plant hormone signal transduction, transcription factors, protein ubiquitination, detoxification, phenylpropanoid biosynthesis, as well as some other important defence-related pathways were found to be differentially expressed and might play a role in regulating these pathways. Although the mechanisms underlying how these genes and defence-related pathways affect or regulate the response of passion fruit to viral pathogen still require further research in future studies, the knowledge obtained from this study will serve as a useful genetic resource to facilitate further investigations of passion fruit response to virus infection, and provide many possible directions for further research about the interactions between passion fruit and viral pathogens. Besides, the transcriptome data generated here also will help guide further research to develop novel strategies for disease management in passion fruit.

## Supporting information

S1 Fig The length distribution of transcripts. (TIF)

S2 Fig The length distribution of unigenes. (TIF)

S3 Fig Unigene COG annotations. (TIF)

S4 Fig Unigene GO annotations. (TIF)

S5 Fig Unigene KEGG annotations. (TIF)

S6 Fig Phenylpropanoid biosynthesis pathway map ko00940. (TIF)

S7 Fig. (a) The activity of PAL; (b) Expression profiling of DEGs were assigned to the “detoxification” GO term; (c) Expression profiling of DEGs encoding wound-induced protein; (d) Expression profiling of DEGs encoding pathogenesis-related proteins; (e) Expression profiling of DEGs encoding RNA-dependent RNA polymerases (TIF)

S8 Fig. (a) Electrolyte leakage; (b) TBARS content; (c) Paraffin section. The scale bars indicate 100 μm (TIF)

S9 Fig. (a-d) The activities of APX, CAT, POD and SOD. DEGs involved in ROS response (TIF)

S10 Fig Comparison of the relative expression levels change of nine selected DEGs by RNA-seq and qRT-PCR. Left verticaL axis coordinate is FPKM of RNA-seq; Right vertical axis coordinate is relative expression level of qRT-PCR.
(TIF)

S11 Fig Zeatin biosynthesis pathway map ko00908. (TIF)

S1 Data List of all up-regulated DEGs in CK vs. CMV. (XLS)

S2 Data List of all down-regulated DEGs in CK vs. CMV. (XLS)

S3 Data GO enrichment of DEGs in CK vs. CMV. (XLS)

S4 Data KOG classification of DEGs in CK vs. CMV. (XLS)

S5 Data Top twenty KEGG pathway in CK vs. CMV. (XLS)

S1 Table Passion fruit gene primers be used in this study. (DOCX)

S2 Table Statistics of clean data in all samples. (DOCX)

S3 Table Statistic of passion fruit transcripts length. (DOCX)

S4 Table Statistic of passion fruit unigenes length. (DOCX)

S5 Table Statistic of passion fruit unigenes function annotation. (DOCX)
