# Supplementary figures and images for: Transcriptome analysis of yellow passion fruit in response to cucumber mosaic virus infection
Source: PLoS One. 2021 Feb 24;16(2):e0247127. doi: 10.1371/journal.pone.0247127 (PMC7904197; doi:10.1371/journal.pone.0247127)

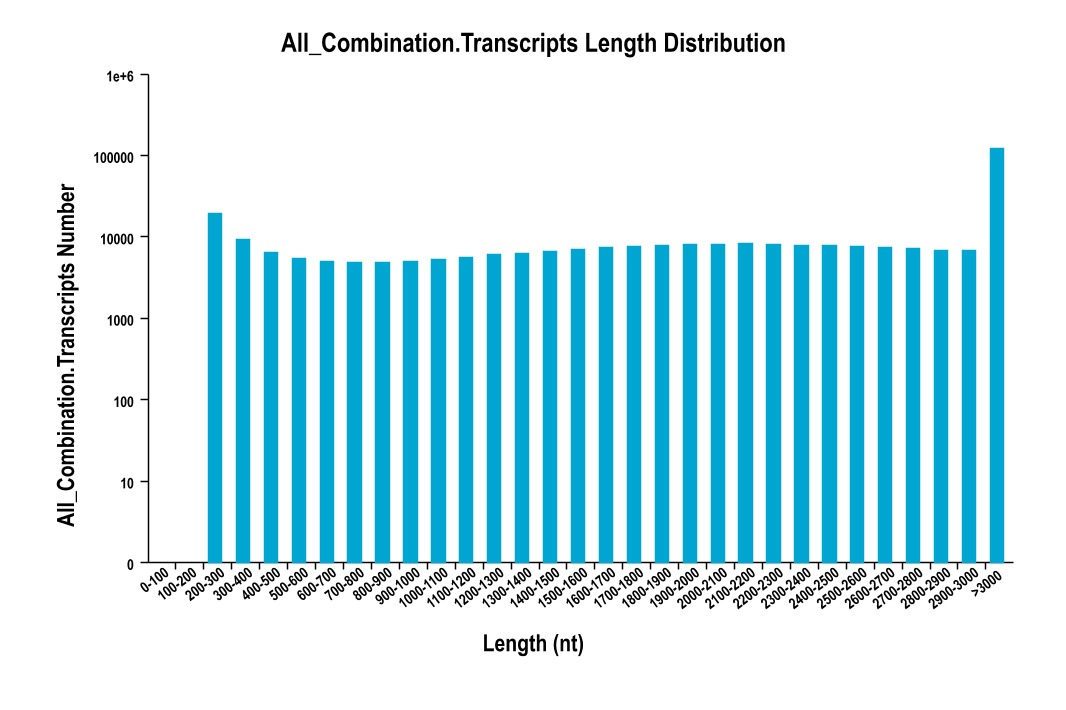

Supplement: S1 Fig — (TIF) [file pone.0247127.s001.tif]

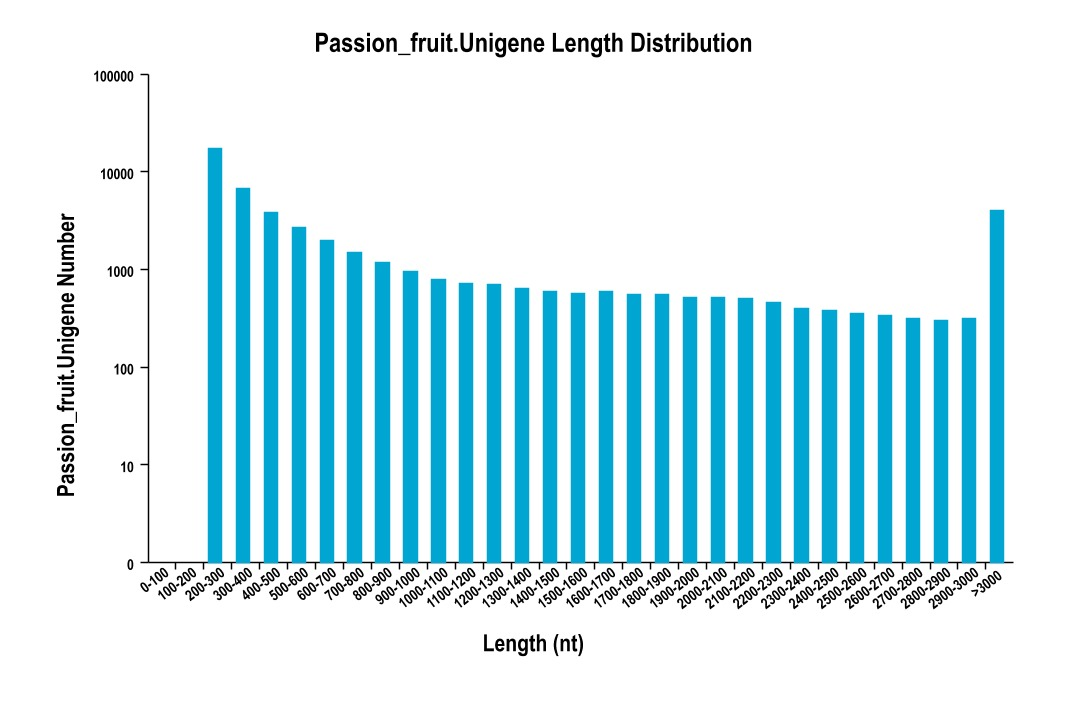

Supplement: S2 Fig — (TIF) [file pone.0247127.s002.tif]

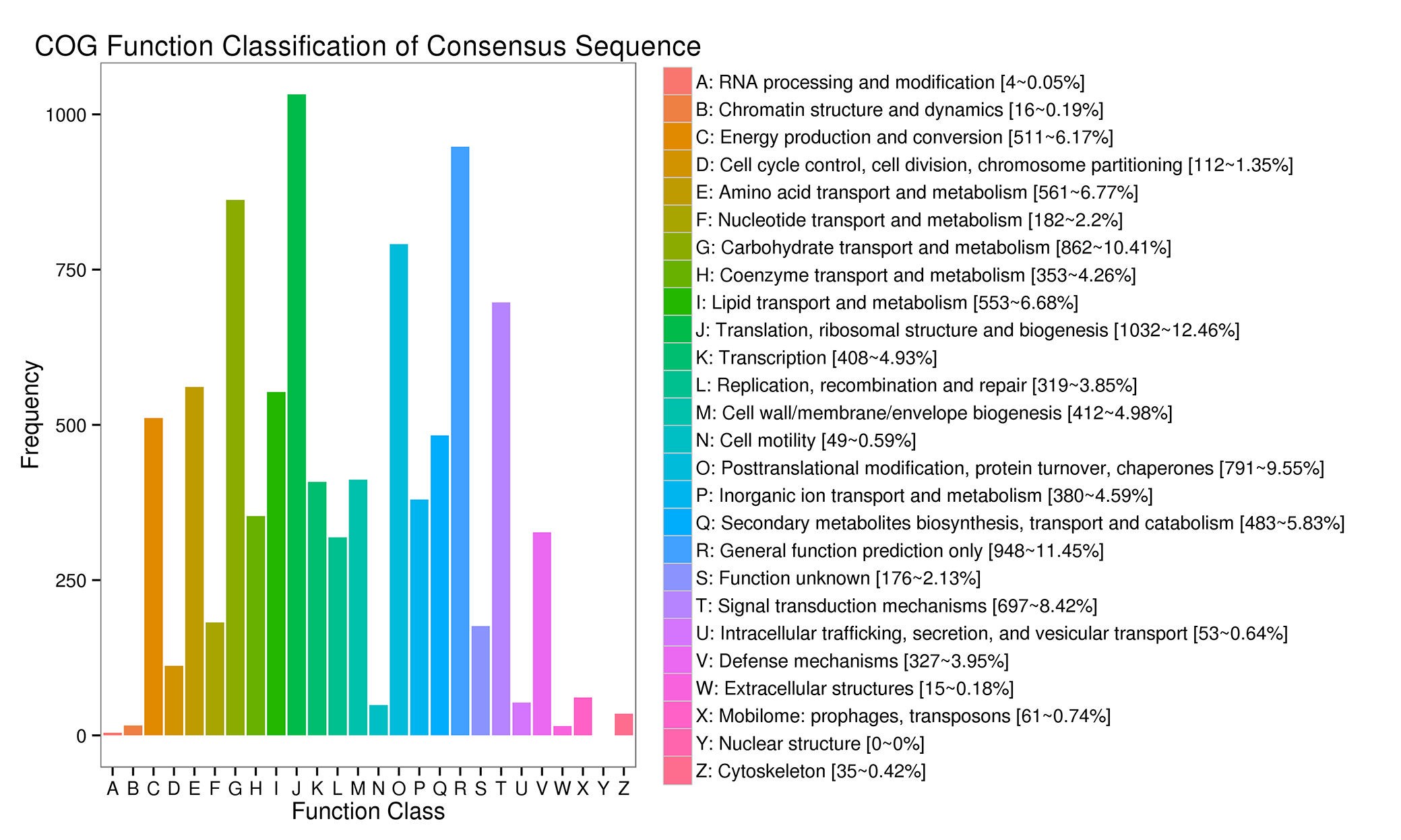

Supplement: S3 Fig — (TIF) [file pone.0247127.s003.tif]

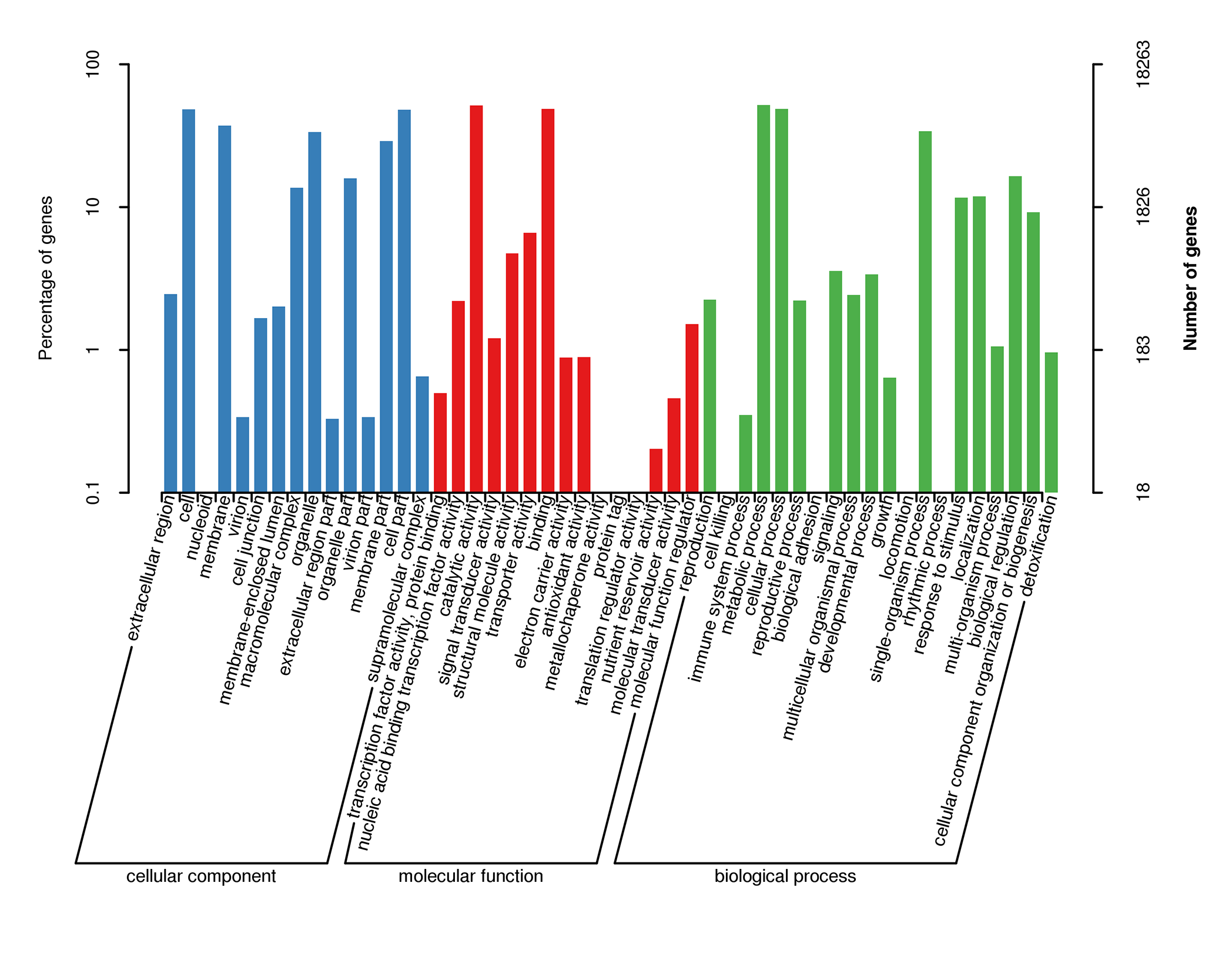

Supplement: S4 Fig — (TIF) [file pone.0247127.s004.tif]

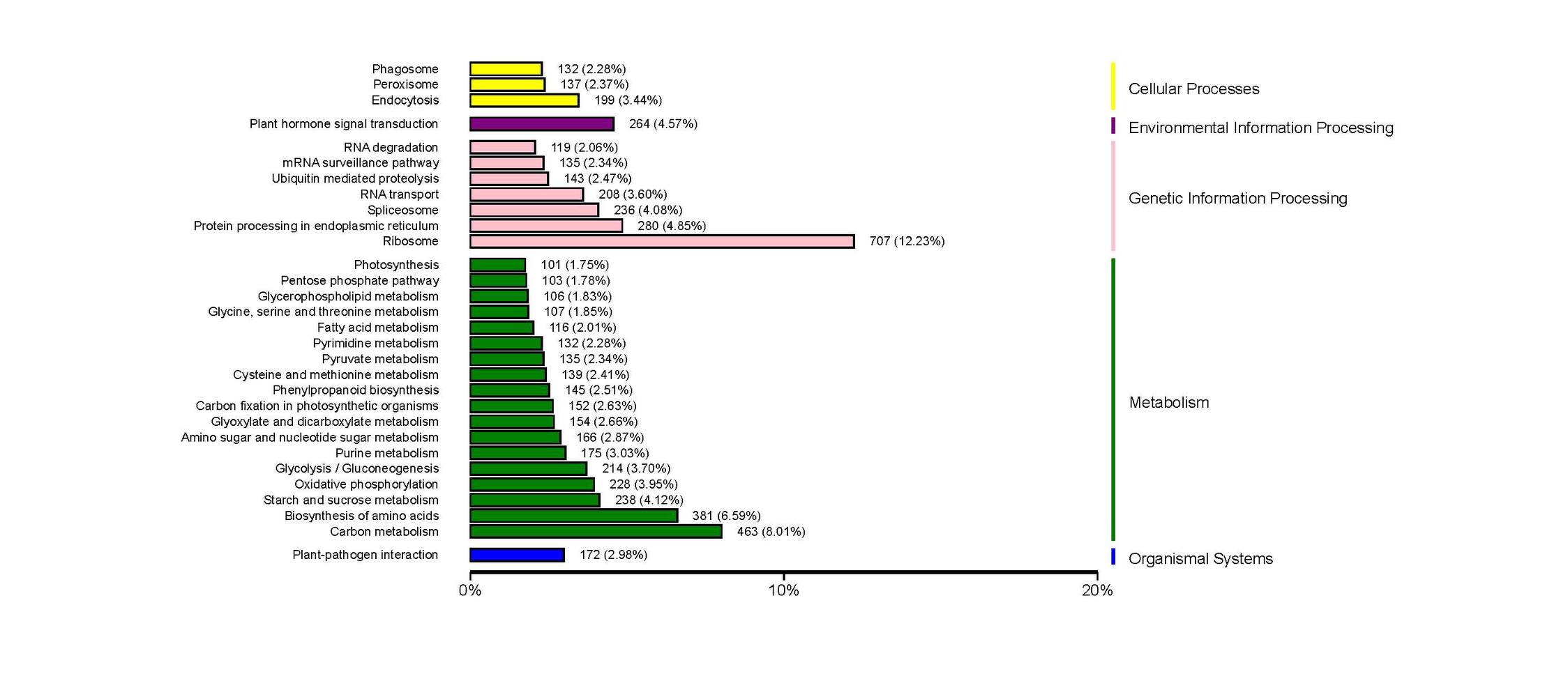

Supplement: S5 Fig — (TIF) [file pone.0247127.s005.tif]

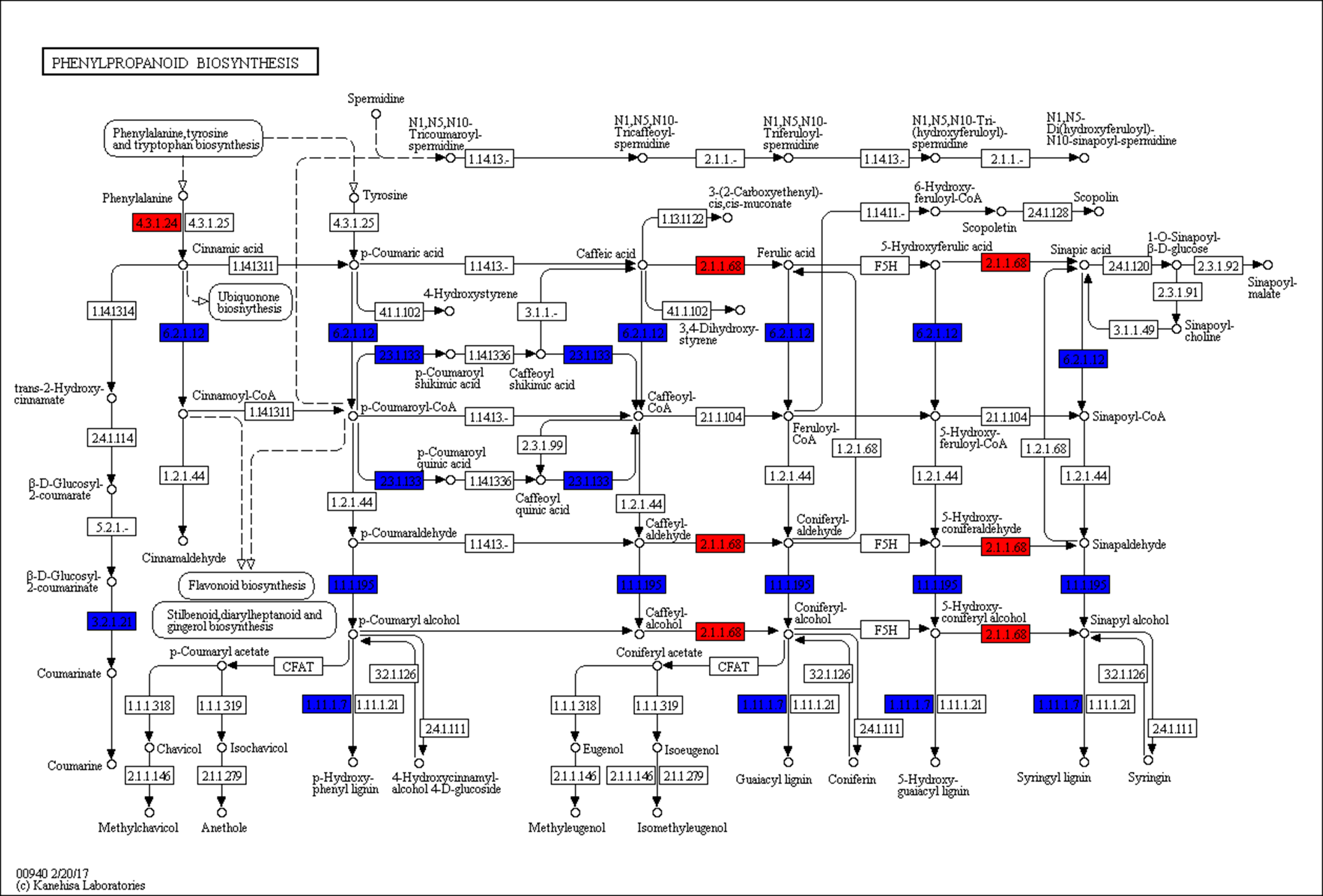

Supplement: S6 Fig — (TIF) [file pone.0247127.s006.tif]

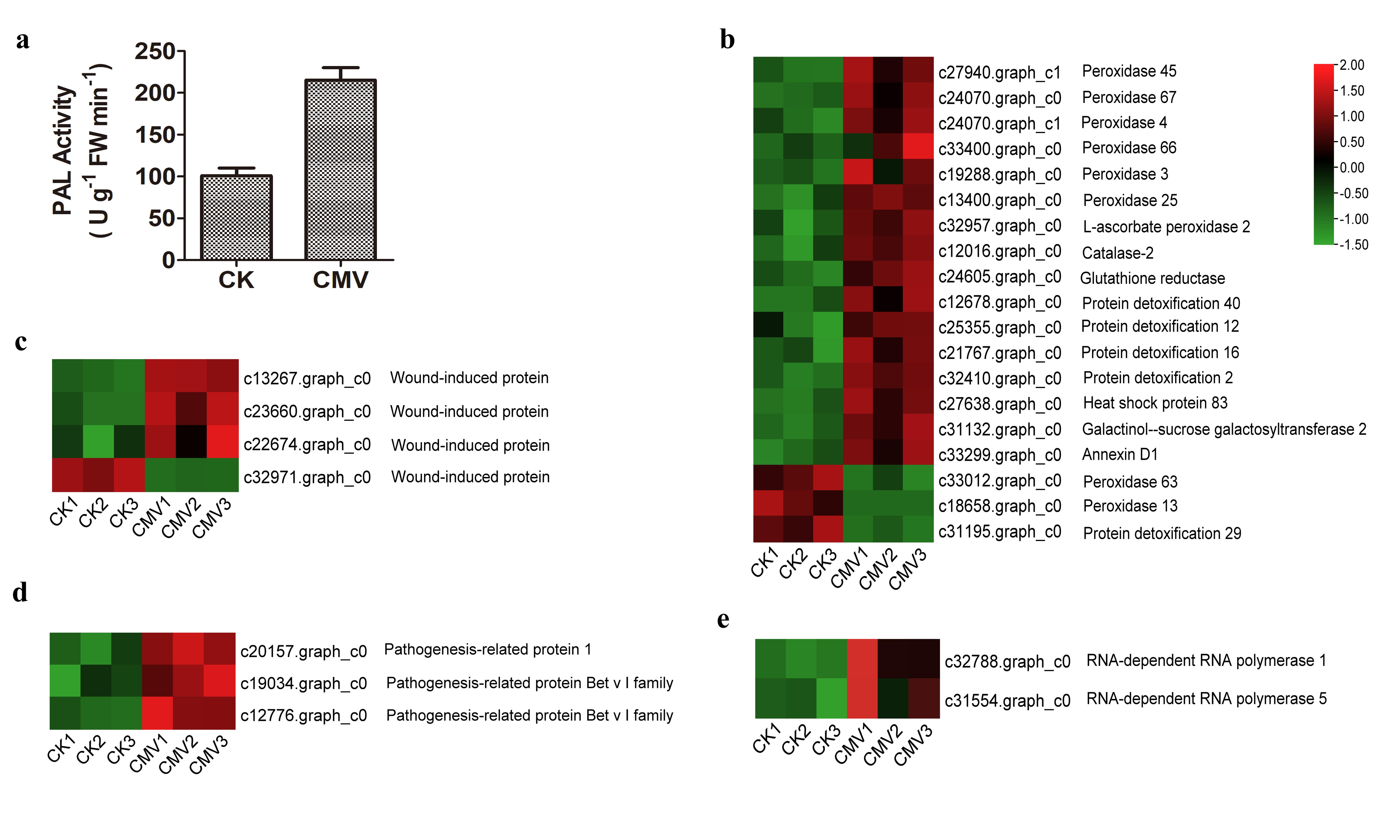

Supplement: S7 Fig. (a) The activity of PAL; (b) Expression profiling of DEGs were assigned to the “detoxification” GO term; (c) Expression profiling of DEGs encoding wound-induced protein; (d) Expression profiling of DEGs encoding pathogenesis-related proteins; (e) Expression profiling of DEGs encoding RNA-dep — (TIF) [file pone.0247127.s007.tif]

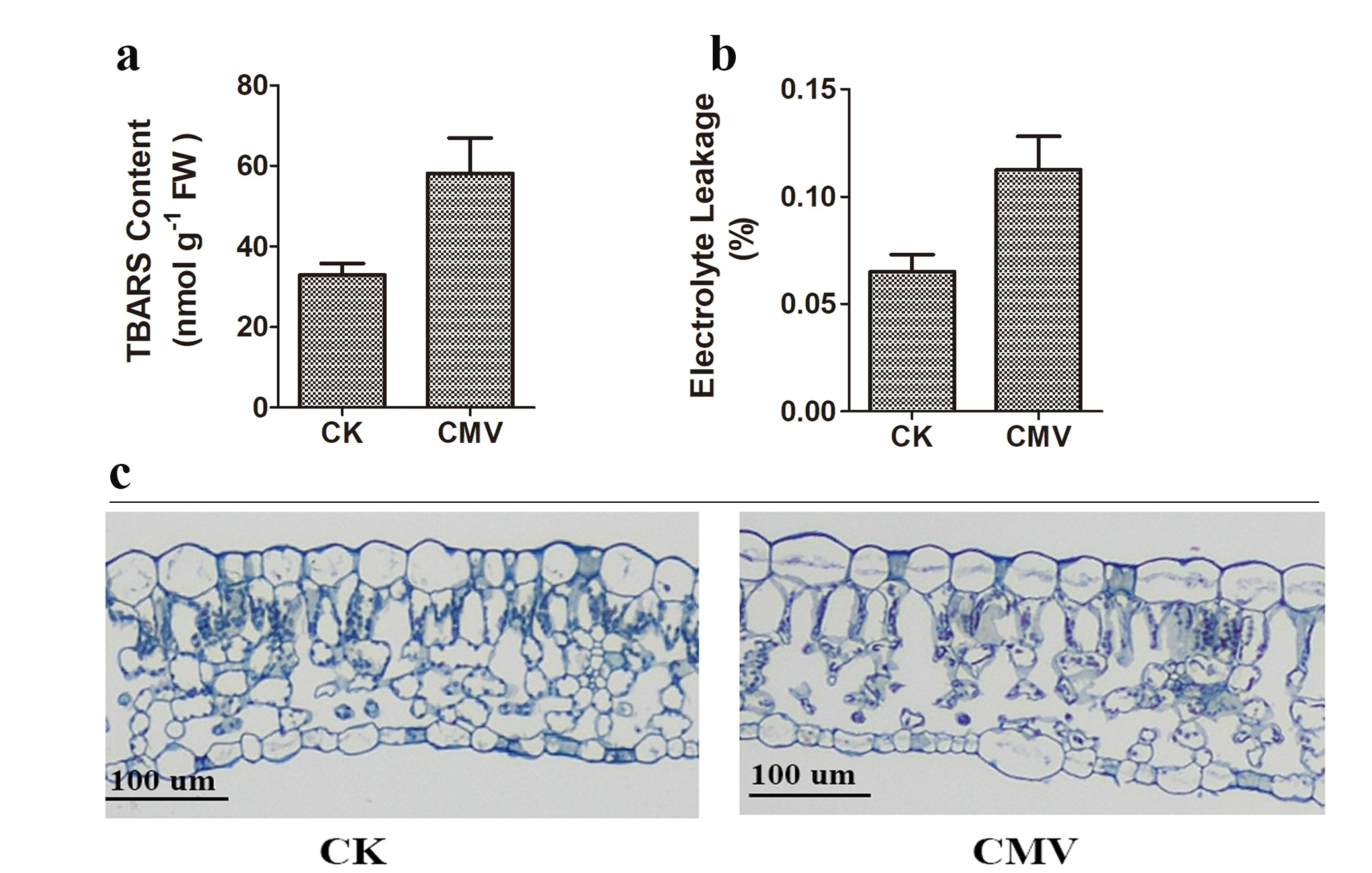

Supplement: S8 Fig. (a) Electrolyte leakage; (b) TBARS content; (c) Paraffin section. The scale bars indicate 100 μm — (TIF) [file pone.0247127.s008.tif]

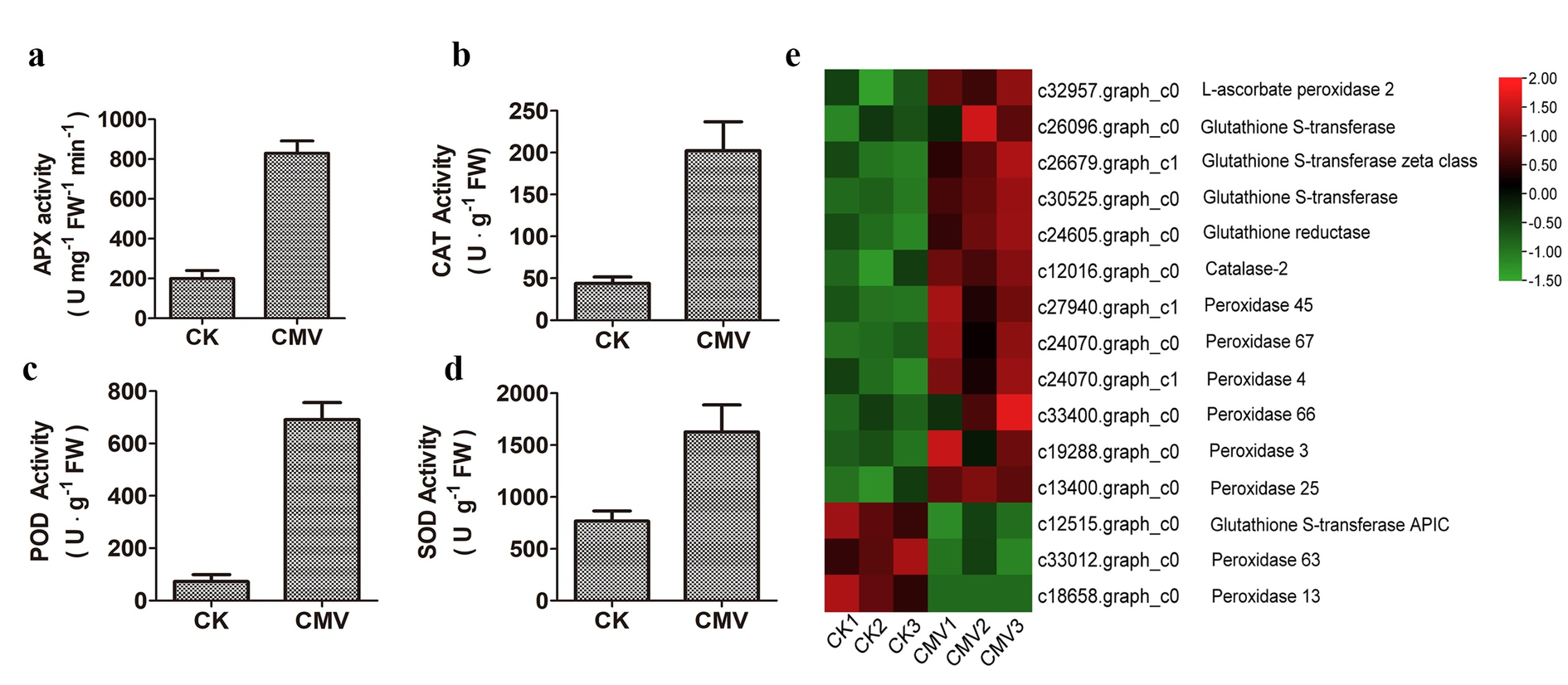

Supplement: S9 Fig. (a-d) The activities of APX, CAT, POD and SOD. DEGs involved in ROS response — (TIF) [file pone.0247127.s009.tif]

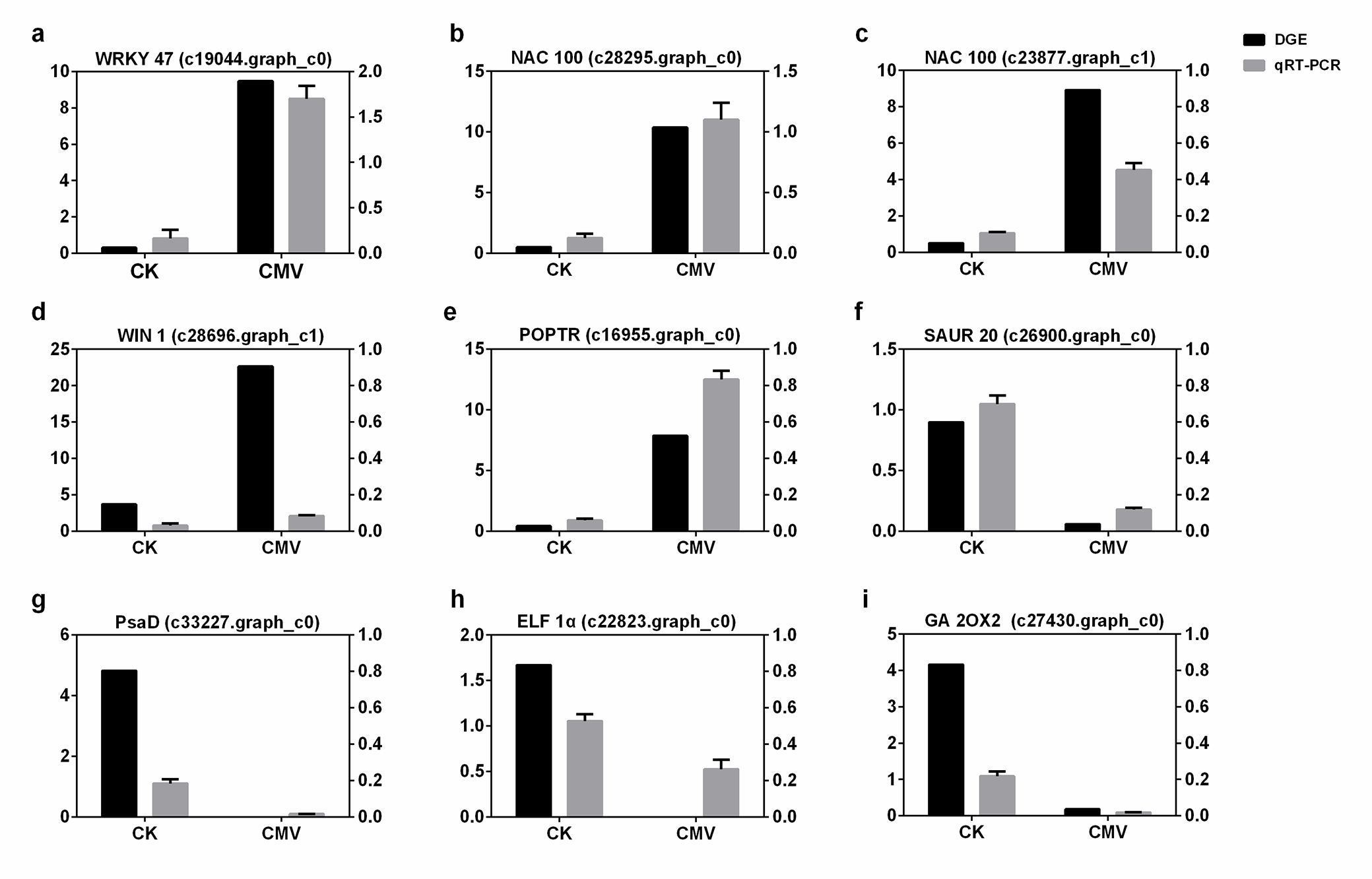

Supplement: S10 Fig — Left verticaL axis coordinate is FPKM of RNA-seq; Right vertical axis coordinate is relative expression level of qRT-PCR. (TIF) [file pone.0247127.s010.tif]

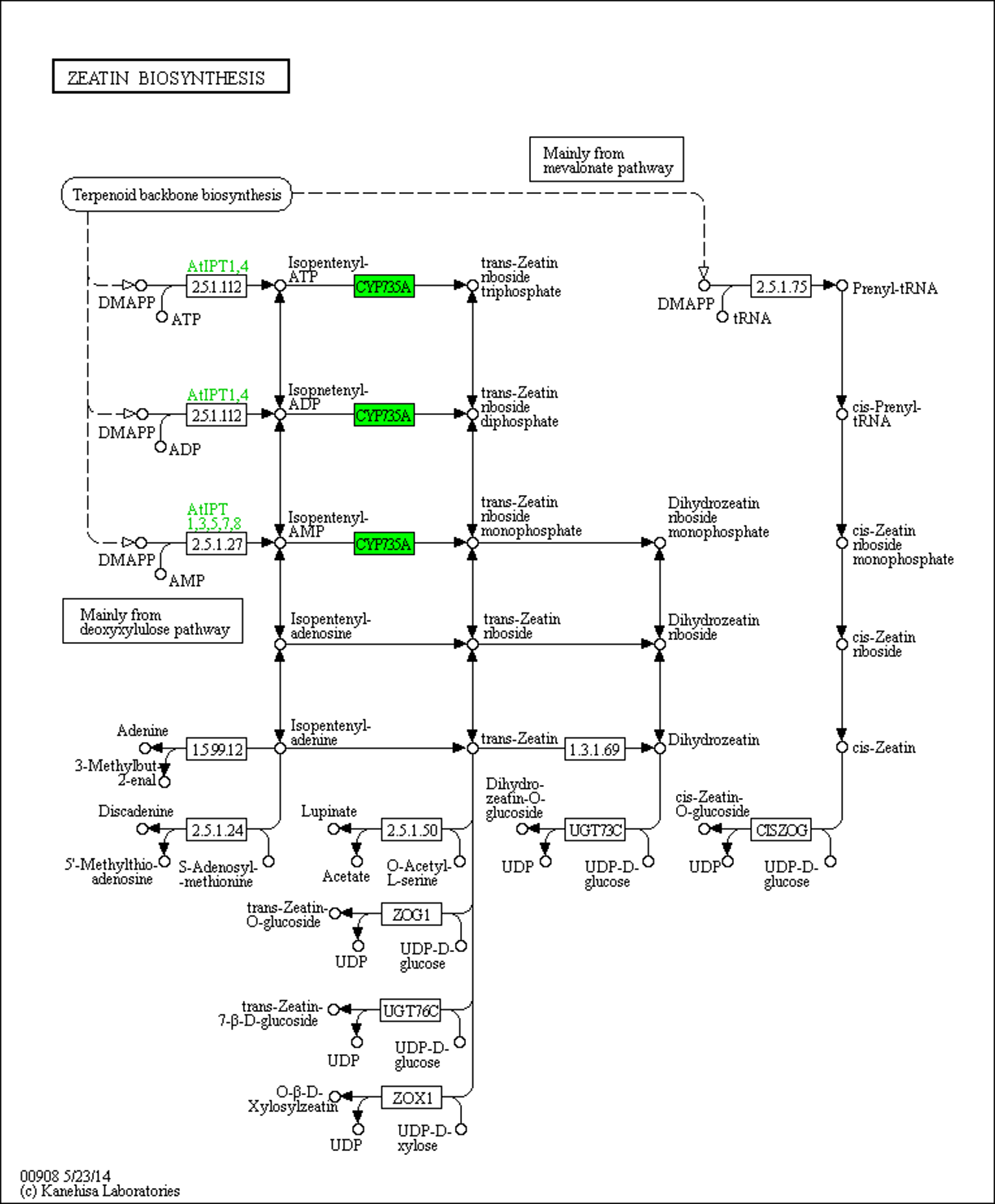

Supplement: S11 Fig — (TIF) [file pone.0247127.s011.tif]
