# Supplementary material for: Transcriptome analysis of yellow passion fruit in response to cucumber mosaic virus infection
Source: PLoS One. 2021 Feb 24;16(2):e0247127. doi: 10.1371/journal.pone.0247127 (PMC7904197; doi:10.1371/journal.pone.0247127)
Supplement: S1 Table — (DOCX) [file pone.0247127.s017.docx]

**S1 Table.** Passion fruit gene primers be used in this study.

| Gene ID | F | R |
| --- | --- | --- |
| c19044.graph_c0 | CCGAGGATAGGAGTATTC | AGGTAGTGTTAGCCATAG |
| c28295.graph_c0 | ACAAGCCTCGTAGAACAT | ACACCAATATCCAGACTCAAT |
| c23877.graph_c1 | ATGTTGAGGATGTTACTTGAA | TTCGTGGCTTGTCATTAC |
| c28696.graph_c1 | AACGCAGTCCATCCATAC | TACCACTTATACAATCCTCAGAAT |
| c16955.graph_c0 | GAAGAGTTGCTGGAAGTC | CACCGCCTTATCAATGAA |
| c26900.graph_c0 | ACTGGTGAATAATCCTGAATGTT | CTTGGTGCTCTGCGATTG |
| c33227.graph_c0 | TACACTTGCTTGCCAGTA | TTTATCCCGAGAAGGTGAA |
| c22823.graph_c0 | CAGGTATGGTTGTCACTT | CGTTGAATCCTACATTGTC |
| c27430.graph_c0 | GGCAACAACCTGAAGTAG | CGTAGTTCCTGTCCATTG |
| c32915.graph_c0 | GTAGGTTGCCATAATCAC | ATTAGAACTGCTTGAACTC |
