# Supplementary material for: Transcriptome analysis of yellow passion fruit in response to cucumber mosaic virus infection
Source: PLoS One. 2021 Feb 24;16(2):e0247127. doi: 10.1371/journal.pone.0247127 (PMC7904197; doi:10.1371/journal.pone.0247127)
Supplement: S2 Table — (DOCX) [file pone.0247127.s018.docx]

**S2 Table.** Statistics of clean data in all samples.

| Sample | Read Number | Base Number | GC Content | %≥Q30 |
| --- | --- | --- | --- | --- |
| CK1 | 29,184,031 | 8,707,509,112 | 46.84% | 95.18% |
| CK2 | 26,439,556 | 7,894,566,008 | 46.98% | 94.83% |
| CK3 | 21,850,221 | 6,509,845,964 | 47.19% | 95.41% |
| CMV1 | 20,492,822 | 6,107,627,884 | 47.10% | 94.80% |
| CMV2 | 21,476,562 | 6,406,083,448 | 46.91% | 94.65% |
| CMV3 | 22,132,110 | 6,607,061,024 | 46.67% | 94.37% |
