# Supplementary material for: Transcriptome analysis of yellow passion fruit in response to cucumber mosaic virus infection
Source: PLoS One. 2021 Feb 24;16(2):e0247127. doi: 10.1371/journal.pone.0247127 (PMC7904197; doi:10.1371/journal.pone.0247127)
Supplement: S3 Table — (DOCX) [file pone.0247127.s019.docx]

**S3 Table.** Statistic of passion fruit transcripts length.

| **All combination transcripts length** | **Total Number** | **Percentage** |
| --- | --- | --- |
| 200-300 | 19536 | 6.02% |
| 300-500 | 15675 | 4.83% |
| 500-1000 | 24910 | 7.68% |
| 1000-2000 | 67521 | 20.81% |
| 2000+ | 196876 | 60.67% |
| Total Number | 324518 |  |
| Total Length | 872825036 |  |
| N50 Length | 3583 |  |
| Mean Length | 2689.60438558108 |  |
