# Supplementary material for: Transcriptome analysis of yellow passion fruit in response to cucumber mosaic virus infection
Source: PLoS One. 2021 Feb 24;16(2):e0247127. doi: 10.1371/journal.pone.0247127 (PMC7904197; doi:10.1371/journal.pone.0247127)
Supplement: S4 Table — (DOCX) [file pone.0247127.s020.docx]

**S4 Table.** Statistic of passion fruit unigenes length.

| **Passion fruit unigenes length** | **Total Number** | **Percentage** |
| --- | --- | --- |
| 200-300 | 17,100 | 34.10519% |
| 300-500 | 10,613 | 21.16716% |
| 500-1000 | 8,270 | 16.49415% |
| 1000-2000 | 6,222 | 12.4095% |
| 2000+ | 7,934 | 15.82401% |
| Total Number | 50,139 |  |
| Total Length | 50,470,866 |  |
| N50 Length | 2,202 |  |
| Mean Length | 1,006.618919 |  |
