# Supplementary material for: Transcriptome analysis of yellow passion fruit in response to cucumber mosaic virus infection
Source: PLoS One. 2021 Feb 24;16(2):e0247127. doi: 10.1371/journal.pone.0247127 (PMC7904197; doi:10.1371/journal.pone.0247127)
Supplement: S5 Table — (DOCX) [file pone.0247127.s021.docx]

**S5 Table.** Statistic of passion fruit unigenes function annotation.

|  | **Annotated Number** | **Percentage (%)** |
| --- | --- | --- |
| **COG_Annotation** | 8389 | 16.73% |
| **GO_Annotation** | 18263 | 36.42% |
| **KEGG_Annotation** | 10229 | 20.4% |
| **KOG_Annotation** | 15240 | 30.4% |
| **Pfam_Annotation** | 17974 | 35.85% |
| **Swissprot_Annotation** | 18118 | 36.14% |
| **eggNOG_Annotation** | 25347 | 50.55% |
| **NR_Annotation** | 26203 | 52.33% |
| **All_Annotated** | 27203 | 54.26% |
| **Total Unigenes** | 50139 | 100 |
